# Targeting Epigenetic Regulation of miR-34a for Treatment of Pancreatic Cancer by Inhibition of Pancreatic Cancer Stem Cells

**DOI:** 10.1371/journal.pone.0024099

**Published:** 2011-08-31

**Authors:** Dara Nalls, Su-Ni Tang, Marianna Rodova, Rakesh K. Srivastava, Sharmila Shankar

**Affiliations:** 1 Departments of Pathology and Laboratory Medicine, The University of Kansas Medical Center, Kansas City, Kansas, United States of America; 2 Department of Pharmacology, Toxicology and Therapeutics, The University of Kansas Medical Center, Kansas City, Kansas, United States of America; 3 Department of Medicine, The University of Kansas Medical Center, Kansas City, Kansas, United States of America; 4 The University of Kansas Cancer Center, The University of Kansas Medical Center, Kansas City, Kansas, United States of America; University of Nebraska Medical Center, United States of America

## Abstract

**Background:**

MicroRNA-34a (miR-34a) is a transcriptional target of p53 and is down-regulated in pancreatic cancer. This study aimed to investigate the functional significance of miR-34a in pancreatic cancer progression through its epigenetic restoration with chromatin modulators, demethylating agent 5-Aza-2′-deoxycytidine (5-Aza-dC) and HDAC inhibitor Vorinostat (SAHA).

**Methodology/Principal Findings:**

Re-expression of miR-34a in human pancreatic cancer stem cells (CSCs) and in human pancreatic cancer cell lines upon treatment with 5-Aza-dC and SAHA strongly inhibited the cell proliferation, cell cycle progression, self-renewal, epithelial to mesenchymal transition (EMT) and invasion. In pancreatic CSCs, modulation of miR-34a induced apoptosis by activating caspase-3/7. Treatment of pancreatic CSCs with the chromatin-modulating agents resulted in the inhibition of Bcl-2, CDK6 and SIRT1, which are the putative targets of miR-34a. MiR-34a upregulation by these agents also induced acetylated p53, p21^WAF1^, p27^KIP1^ and PUMA in pancreatic CSCs. Inhibition of miR-34a by antagomiR abrogates the effects of 5-Aza-dC and SAHA, suggesting that 5-Aza-dC and SAHA regulate stem cell characteristics through miR-34a. In CSCs, SAHA inhibited Notch pathway, suggesting its suppression may contribute to inhibition of the self-renewal capacity and induction of apoptosis. Interestingly, treatment of pancreatic CSCs with SAHA resulted in the inhibition of EMT with the transcriptional up-regulation of E-Cadherin and down-regulation of N-Cadherin. Expression of EMT inducers (Zeb-1, Snail and Slug) was inhibited in CSCs upon treatment with SAHA. 5-Aza-dC and SAHA also retard *in vitro* migration and invasion of CSCs.

**Conclusions:**

The present study thus demonstrates the role of miR-34a as a critical regulator of pancreatic cancer progression by the regulating CSC characteristics. The restoration of its expression by 5-Aza-dC and SAHA in CSCs will not only provide mechanistic insight and therapeutic targets for pancreatic cancer but also promising reagents to boost patient response to existing chemotherapies or as a standalone cancer drug by eliminating the CSC characteristics.

## Introduction

Pancreatic cancer is the fourth leading cause of cancer death in United States and is a devastating invasive disease and one of the most aggressive cancers [1]. The poor prognosis of pancreatic adenocarcinoma is attributed to its late presentation, lack of accurate biomarkers for early diagnosis for the possibility of curative resection as well as the propensity of early metastasis. Therefore, novel diagnostic modalities for early diagnosis and new therapeutic strategies are urgently needed.

MicroRNAs (miRNAs) are endogenous, noncoding small RNAs 19–25 nucleotides in length, which are now recognized as crucial post transcriptional regulators of gene expression [2], [3], [4]. The ability of miRNAs to regulate multiple genes conform them to play important roles in biological processes that effect tumor progression including migration, invasion, epithelial to mesenchymal transition (EMT) and metastasis [5], [6], [7], [8], [9], [10]. MiRNAs are very promising as early biomarkers, prognostic indicators and mechanism based therapeutic targets for anticancer treatments [11], [12], [13], [14], [15] because their aberrant expressions are linked to cancer stem cell (CSC) deregulation and thus oncogenesis [16].

Cancer stem cells give rise to the tumor bulk through continuous self-renewal and differentiation [17]. Pancreatic CSCs are highly tumorigenic and self renewing sub-population that express the cell surface marker CD133+/CD44+/CD24+/ESA+ [17], [18], [19]. Strategies are being developed towards the targeted destruction of these tumor stem cells while sparing the physiological stem cells, which may lead to marked improvement in patient outcome. By altering the expression of specific miRNAs that may play an important role in pancreatic CSC renewal and may thus contribute to the development of pancreatic carcinoma, would help achieve a selective and targeted elimination of the pancreatic CSCs. Therefore, understanding the mechanisms that regulate self-renewal is of greatest importance for discovery of anticancer drugs targeting CSCs.

TP53 is an important tumor suppressor gene whose biological effects are largely due to its function as a transcriptional regulator [20]. TP53 mutations are relatively common and occur in up to 70% of cases with pancreatic adenocarcinoma and are most commonly seen in poorly differentiated tumors [21], [22], [23], [24], [25]. The tumor suppressor TP53 has been identified as a transcriptional regulator of miR-34a and several recent studies have implicated the miR-34 family of miRNAs in the p53 tumor suppressor network [20], [26]. miR-34a is highly expressed in normal tissues, like testis, lung, adrenal gland and spleen, although its physiological function is unknown. miR-34a is localized on human chromosome 1p36, which is a region associated with a variety of cancers, and has been shown to be down-regulated in pancreatic cancer [20]. Reduced expression of miR-34a in pancreatic cancer could be a resultant of either transcriptional regulation due to p53 mutations as these are very frequent [22] or through epigenetic silencing. Therefore, understanding the contributions of these mechanisms in the down regulation of miR-34a in pancreatic cancer, which may contribute to the malignancy of the pancreas, could be of relevance.

MiR-34a acts as a suppressor of neuroblastoma tumorigenesis by targeting the mRNA encoding E2F3 and reducing E2F3 protein levels [27]. It has also been shown to be hyper-methylated in breast, ovarian, colon, lung and haematological malignancies and downregulate CDK6 translation thereby demonstrating the tumor suppressor role of miR-34a [28], [29], [30]. The miR-34a responsive genes are highly enriched for those that regulate cell-cycle progression, cellular proliferation, apoptosis, DNA repair, and angiogenesis thereby providing a functional basis for the epigenetic inactivation of this miRNA in pancreatic cancer [31].

It is well established that many tumor suppressor genes in human cancers are silenced by promoter methylation accompanied by chromatin alterations due to recruitment of histone deactylases by proteins binding to methylated CpG-residues. These epigenetic markers associated with the silencing of tumor suppressor genes may contribute in the tumor progression and can be therapeutically reversible, serving as targets for pancreatic cancer treatment. Based on this premise, we quantified the expression of miR-34a in human pancreatic CSCs and pancreatic cancer cell lines irrespective of the p53 mutation status, compared to normal pancreatic ductal epithelial cells using TaqMan miRNA assays.

Notch signals are known to affect stem cell selfrenewal and differentiation, and have been suggested to play a role during pancreatic carcinogenesis [32], [33]. Several members of the Notch signaling pathway are expressed in the pancreas [32]. The Notch receptors are activated by Delta and Serrate/Jagged ligands [34], which promote proteolytic cleavage and the release of the Notch intracellular domain (ICD). The activated Notch-ICD translocates into the nucleus and interacts with the transcription factor CSL/RBP-Jκ and the co-activator Mastermind (MAM) to activate target genes such as Hes1 and Hes5 [35], [36], and Hes expression can therefore be used as a read out for Notch activity [37].

Our present studies have revealed that expression levels of miR-34a were significantly reduced in pancreatic CSCs and pancreatic cancer tumor cells independent of their p53 mutational status, compared to normal pancreatic ductal epithelial cells. This lead us to hypothesize that miR-34a, thus may be epigenetically silenced in pancreatic cancer. Reduction of miR-34a gene methylation and altered acetylation pattern by the use of chromatin-modifying agents resulted in concomitant reactivation of miR-34a expression. Expression of miR-34a in pancreatic CSCs and cell lines was significantly induced on treatment with chromatin modifiers, demethylating agent 5-aza-2′-deoxycytidine (5-Aza-dC) or the histone deacetylase inhibitor, SAHA (vorionostat). 5-Aza-dC and SAHA also inhibited the growth and induced apoptosis in pancreatic CSCs. Further, to understand the functional role of miR-34a in the regulation of pancreatic cancer progression the effect of 5-Aza-dC and SAHA on the protein expression of putative targets of miR-34a in pancreatic CSCs was examined. 5-Aza-dC inhibited the expression of cyclin D1 and CDK4 and on the contrary induced the expression of p27^/KIP1^, and these effects were abrogated in the presence of miR34a antagonist. Similarly, SAHA down-regulated the expression of SIRT1, cyclin D1, survivin, Bcl-2, VEGF and CDK6, and up-regulated the expression of p21 and PUMA in a dose-dependent manner. Further, treatment of pancreatic CSCs with the chromatin modifiers resulted in the inhibition of self-renewal, EMT, migration and invasion of pancreatic CSCs *in vitro*. Modulation of the expression of miR-34a by SAHA inhibited the mRNA expression of various components of the Notch pathway, thus suggesting that miR-34a may be involved in pancreatic CSC self-renewal. Zeb-1, Snail and Slug transcriptional expression was significantly decreased in human pancreatic CSCs and pancreatic cancer cell lines upon treatment with SAHA. Our results demonstrate that miR-34a might play an important role in the regulation of pancreatic tumorigenesis. This is thus far, the first demonstration of inhibition of pancreatic CSC characteristics by epigenetic modulation of miR-34a by therapeutic intervention using 5-Aza-dC and SAHA. Therefore, restoration of miR-34a expression by 5-Aza-dC and SAHA in pancreatic CSCs will provide not only unique tools for the investigation of miRNA function, but also promising reagent to boost patient response to existing chemotherapies or as standalone cancer drug.

## Results

### Expression and restoration miR-34a in pancreatic CSCs and cell lines

**Figure 1:**
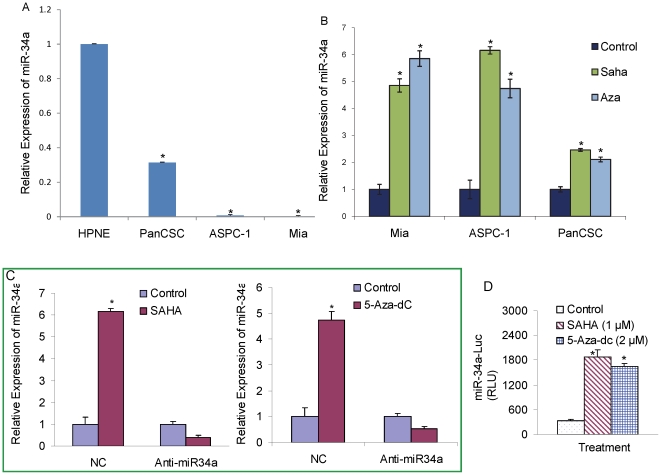
Restoration of the expression of miR-34a in pancreatic cancer cells. (A) Relative expression of miR-34a was quantified in human pancreatic cancer cell lines ASPC-1(p53wt), MiaPACA-2 (p53mutant), human pancreatic cancer stem cells (PanCSC) and human pancreatic normal ductal epithelial cells (HPNE). (B) Restoration of miR34a by SAHA and Aza-5dC. Pancreatic cancer cell lines ASPC-1(p53wt), MiaPACA-2 (p53mutant) and pancreatic cancer stem cells were treated with SAHA (3 µM) or Aza-5dC (4 µM) for 24 h. The expression of miR-34a was quantified using quantitative reverse transcriptase polymerase chain reaction (RT-PCR-Taqman) and Taqman Real Time Assays, and normalized to RNU48 expression. Data represent mean ± SD. *  =  significantly different from control, P<0.05. (C), Anti-miR34a inhibits the ability of SAHA and Aza-5dC to restore miR34a. Pancreatic CSCs were transiently transfected with either negative control (scrambled) or anti-miR34a oligonucleotide, and treated with SAHA (3 µM) or Aza-5dC (4 µM) for 24 h. RNA was extracted to measure the expression of miR34a by q-RT-PCR as described above. NC  =  negative control. (D), miR34a-Luciferase reporter activity. Pancreatic CSCs were transfected with either negative control (scrambled) or anti-miR34a oligonucleotides along with miR34a-Luc construct, and treated with either SAHA (1 µM) or Aza-5dC (2 µM) for 24 h. Luciferase activity was measured as per manufacturer's instructions (Promega). Data represent mean ± SD. *  =  significantly different from control, P<0.05.

We first measured the expression of miR-34a in human pancreatic CSCs derived from primary tumors and pancreatic cancer cell lines [ASPC-1 (p53wt) and MiaPaCa-2 (p53mutant)] and compared with normal pancreatic ductal epithelial cells using quantitative reverse transcriptase polymerase chain reaction (RT-PCR-Taqman) and Taqman Real Time Assays. The comparative Ct (ΔΔCt) method was used to determine the expression fold change of miR-34a in pancreatic cancer cells compared to normal pancreatic epithelial cells. Total RNA input was normalized based on the Ct values obtained for RNU48 used as endogenous control. Consistent with previous studies in pancreatic cancer [20], we observed a global decrease in the expression of miR-34a in pancreatic CSCs and cell lines independent of their p53 mutational status relative to non-neoplastic pancreatic epithelial cells (Fig. 1A).

Mir-34a is closely associated with a large CpG island suggesting that it may be epigenetically silenced in pancreatic cancer. To prove this hypothesis, we examined if the reduced expression of miR-34a in pancreatic cancer could be restored upon treatment with a DNA methylation inhibitor, 5-Aza-dc and/or a histone deacetylase inhibitor, SAHA. Interestingly, miR-34a expression in pancreatic CSCs and pancreatic cancer cell lines [ASPC-1 and MiaPaCa-2] increased significantly following treatment with these chromatin-modifiers compared with mock treated controls, as measured by qRT-PCR (Fig. 1B) using Taqman Real Time Assays using comparative Ct (ΔΔCt) method. Total RNA input was normalized based on the Ct values obtained for RNU48. These results demonstrate that 5-Aza-dC and SAHA induces the restoration of miR-34a in pancreatic cancer cells. The data also suggest that epigenetic modification of regulatory sequences in CpG islands and deacetylation of histones may contribute to miR-34a silencing in pancreatic cancer. Further inhibition of miR-34a by antgomiR abrogates the effects of 5-Aza-dC and SAHA in restoration of the expression of miR-34a in pancreatic CSCs suggesting that 5-Aza-dC and SAHA regulate the stem cell characteristic through miR-34a (Fig. 1C).

We next examined the transcriptional regulation of miR-34a in CSCs by miR-34a-luciferase reporter assay (Fig. 1D). SAHA and 5-Aza-dC induced miR-34a-luciferase activity, suggesting a functional role of miR34a in pancreatic CSCs.

### Upregulation of miR-34a inhibits cell proliferation, and induces apoptosis and cell cycle arrest in human pancreatic CSCs

**Figure 2:**
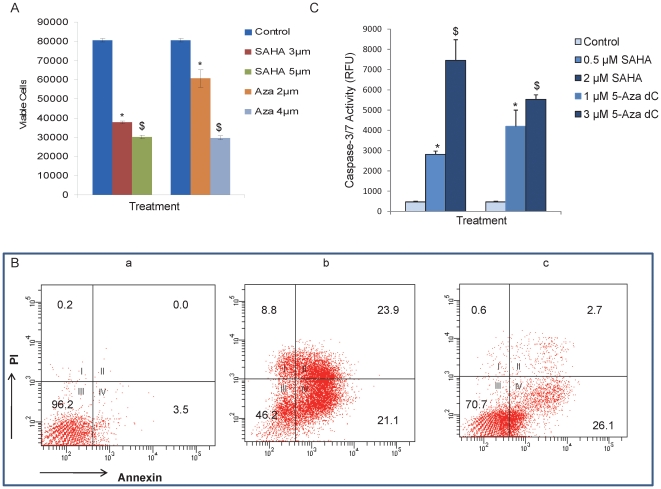
Chromatin modulators inhibit cell viability and promote apoptosis in pancreatic cancer stem cells on restoration of miR-34a. (A), Pancreatic CSCs were treated with SAHA (3 and 5 µM) and 5-Aza-dC (2 and 4 µM) and cell viability was measured at 48 h by staining with trypan blue using Vi-CELL analyzer (Beckman Counter). (B), Pancreatic CSCs were untreated (a) or treated with SAHA (b) or 5Aza-dC (c) for 48 h, and apoptosis was measured by staining with annexin-PI using Accuri Flow Cytometer. (C), Caspase-3/7 activity was measured in pancreatic CSCs treated with SAHA (0.5 and 2 µM) or 5-Aza-dC (1 and 3 µM) for 24 h. Data represent mean ± SD. * and $  =  significantly different from control, P<0.05.

We investigated the effect of 5-Aza-dC and SAHA on the proliferation of pancreatic CSCs by trypan blue staining. 5-Aza-dC and SAHA significantly inhibited cell proliferation in a dose-dependent manner in pancreatic CSCs (Fig. 2A). Further, we examined the effects of these chromatin modulators on the induction of apoptosis and caspase-3/7 activity in pancreatic CSCs by annexin-V and PI staining, and caspase-3/7 activity assay kit, respectively. 5-Aza-dC and SAHA induced apoptosis in CSCs in a dose-dependent manner which was associated with increased caspase-3/7 activity (Fig. 2B and C).

To better understand the biological significance of the restoration of miR-34a, ASPC-1 cells were treated with or without SAHA and its effects on the cell cycle distribution was examined by PI staining using flow cytometry (data not shown). SAHA induced growth arrest in in G2/M phase of the cell cycle at 24 h in AsPC-1 cells. Furthermore, SAHA induced G2/M arrest was abrogated in the presence of miR-34a antagonist relative to the negative control oligonucleotides thus suggesting the involvement of miR-34a in the cell cycle arrest by SAHA (data not shown).

### Effect of 5-Aza-dC and SAHA on the putative targets of the miR-34a in pancreatic CSCs

**Figure 3:**
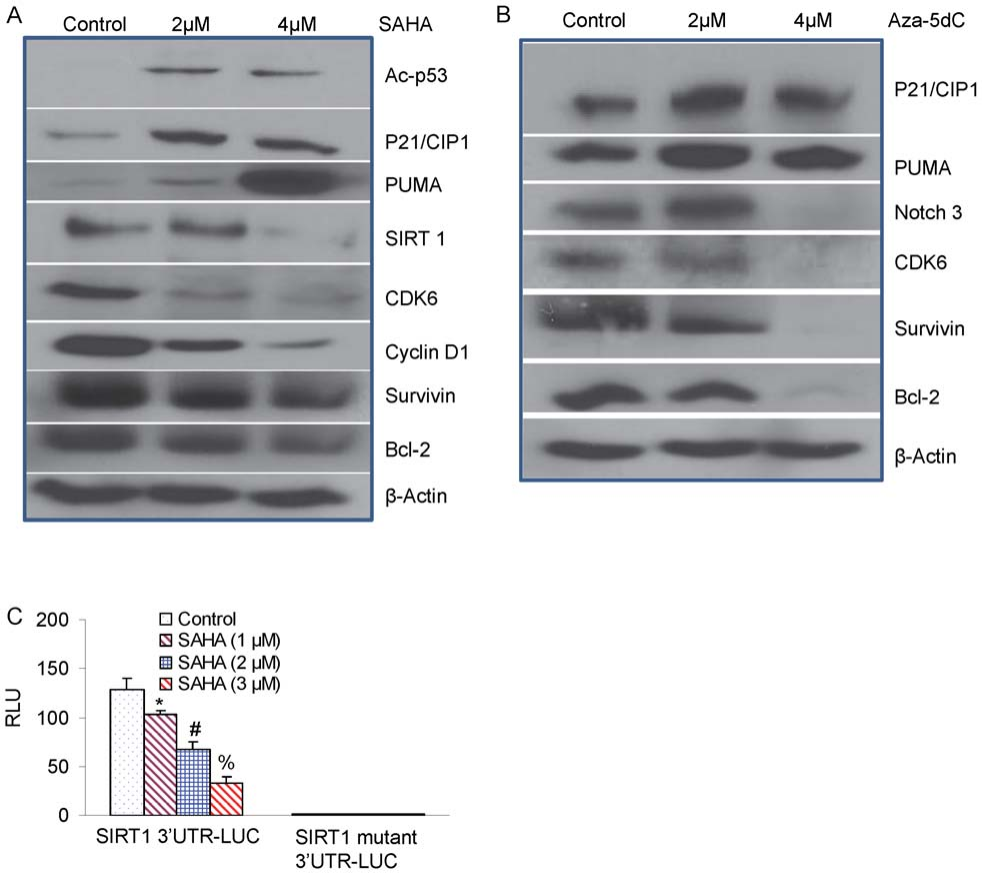
Expression of putative targets of miR-34a. (A), Pancreatic CSCs were treated with or without SAHA for 48 h. The expression of acetylated-p53, p21, PUMA, SIRT1, CDK6, CyclinD1, survivin and Bcl-2 was measured by the Western blot analysis. β-actin was used as a loading control. (B), Pancreatic CSCs were treated with or without 5Aza-dC for 48 h. The expression of p21, PUMA, Notch 3, CDK6, survivin and Bcl-2 was measured by the Western blot analysis. β-actin was used as a loading control. (C), SIRT1 3′UTR-Luciferase activity. Pancreatic CSCs were transduced with either SIRT1 3′UTR-Luc construct or SIRT1 mutant 3′UTR-Luc construct, and treated with SAHA (0–3 µM) for 24 h. Luciferase activity was measured as per manufacturer's instructions (Promega). Data represent mean ± SD. *, # or %  =  significantly different from control, P<0.05.

Since miR-34a is involved in the self renewing capacity and metastasis of pancreatic CSCs, expression of putative targets of miR-34a in pancreatic CSCs on treatment with 5-Aza-dC and SAHA was examined by Western blot analysis (Fig. 3A and B). Our results indicate that 5-Aza-dC and SAHA modulate the protein expression levels of known direct target genes of miR-34a. Interestingly, SAHA inhibited the expression of Cyclin D1 and CDK6 and up regulated the expression of p21/CIP1 in pancreatic CSCs (Fig. 3A). SAHA also inhibited the expression of SIRT1, survivin, Bcl-2, VEGF, and induced the expression of acetylated p53 and PUMA in a dose-dependent manner. Similarly, 5-Aza-dC inhibited the expression of Notch3, CDK6, survivin and Bcl-2, and induced the expression of p21/CIP1 and PUMA in a dose-dependent manner. Since SAHA inhibited the expression of SIRT1 through up-regulation of miR34a, we next examined the effects of SAHA on SIRT1 3′UTR-luciferase reporter activity. SAHA inhibited the SIRT1 3′UTR-Luciferase activity in a dose-dependent manner (Fig. 3C). By comparison, SAHA had no effect on SIRT1 mutant 3′UTR-luciferase activity (containing no functional miR34a binding site). These data suggest that MiR-34a inhibits SIRT1 expression through a miR-34a-binding site within the 3′ UTR of SIRT1.

**Figure 4:**
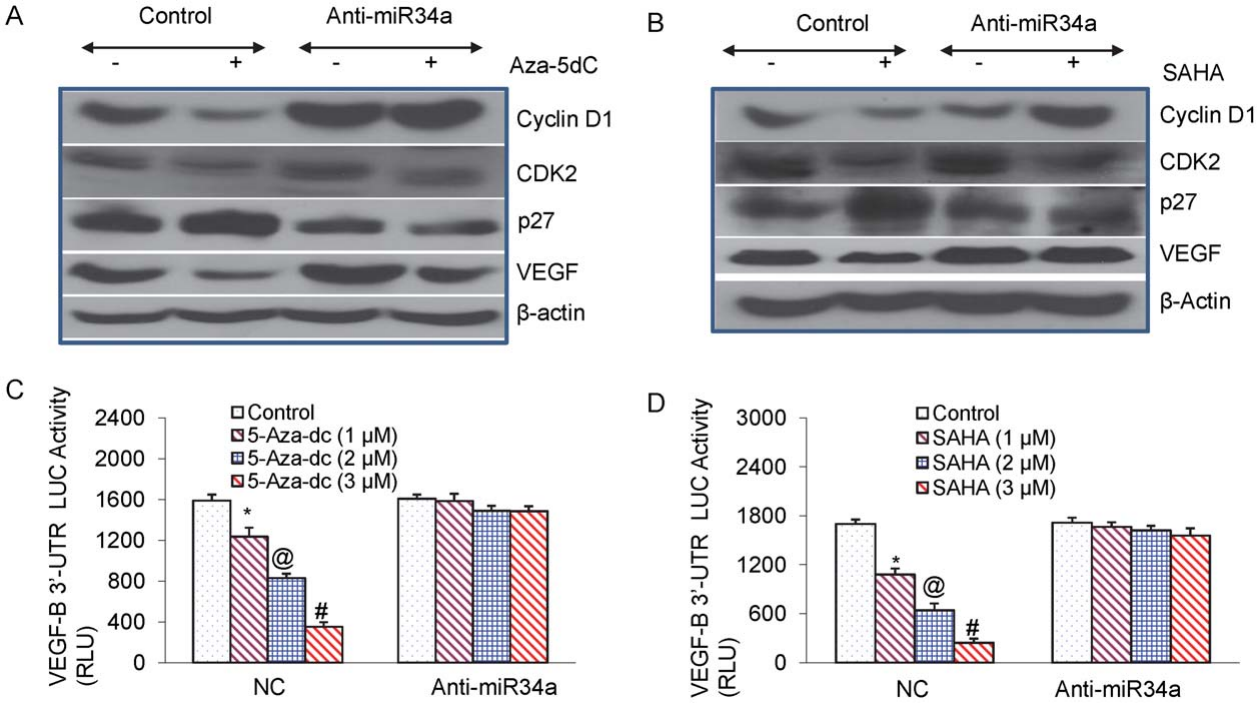
Expression of putative targets of miR-34a. (A), Pancreatic CSCs were transiently transfected with either negative control (scrambled) or anti-miR34a oligonucleotide and treated with Aza-5dC (4 µM) for 48 h. Western blot analysis was performed to measure the expression of cyclin D1, CDK2, p27 and VEGF. β-actin was used as a loading control. (B), Pancreatic CSCs were transiently transfected with either negative control (scrambled) or anti-miR34a oligonucleotide and treated with SAHA (3 µM) for 48 h. Western blot analysis was performed to measure the expression of cyclin D1, CDK2, p27 and VEGF. β-actin was used as a loading control. (C), Regulation of VEGF-B 3′UTR-Luciferase activity by Aza-5dC. Pancreatic CSCs were transfected with either negative control (scrambled) or anti-miR34a oligonucleotides along with VEGF-B 3′UTR-LUC construct, and treated with Aza-5dC (0–3 µM) for 24 h. Luciferase activity was measured as per manufacturer's instructions (Promega). Data represent mean ± SD. *, @ or #  =  significantly different from control, P<0.05. (D), Regulation of VEGF-B 3′UTR-Luciferase activity. Pancreatic CSCs were transfected with either negative control (scrambled) or anti-miR34a oligonucleotides along with VEGF-B 3′UTR-LUC construct, and treated with SAHA (0–3 µM) for 24 h. Luciferase activity was measured as per manufacturer's instructions (Promega). Data represent mean ± SD. *, @, #  =  significantly different from control, P<0.05.

Furthermore, manipulating the expression of miR-34a by using miR-34a antagomiR altered the protein expression of its target genes (Fig. 4A and B). 5-Aza-dC and SAHA inhibits the expression of cell cycle regulatory proteins (Cyclin D1 and CDK2) and VEGF, and up regulates the expression of p27/KIP1 in pancreatic CSCs. Transfection with antagomiR for miR-34a was alone sufficient to abrogate this effect. These data suggest an important and novel mechanism by which 5-Aza-dC and SAHA mediate their effects on cell growth and apoptosis in pancreatic CSCs.

Since 5-Aza-dC inhibited the expression of VEGF through up-regulation of miR34a, we next examined the effects of 5-Aza-dC on VEGF-B 3′UTR-luciferase reporter activity (Fig. 4C). 5-Aza-dC inhibited VEGF-B 3′UTR-luciferase reporter activity in a dose-dependent manner. By comparison, anti-miR34a blocked the inhibitory effects of 5-Aza-dC on luciferase activity. Since SAHA inhibited the expression of VEGF through up-regulation of miR34a, we also examined the effects of SAHA on VEGF-B 3′UTR-luciferase reporter activity. As demonstrated in Fig. 4D, SAHA inhibited the VEGF-B 3′UTR-Luciferase activity in a dose-dependent manner. By comparison, anti-miR34a blocked the inhibitory effects of SAHA on luciferase activity. These data suggest that up-regulation of miR-34a by 5-Aza-dC and SAHA may have functional significance on the regulation of Notch and self-renewal.

### Effect of miR-34a restoration on expression of stem cell renewal genes

**Figure 5:**
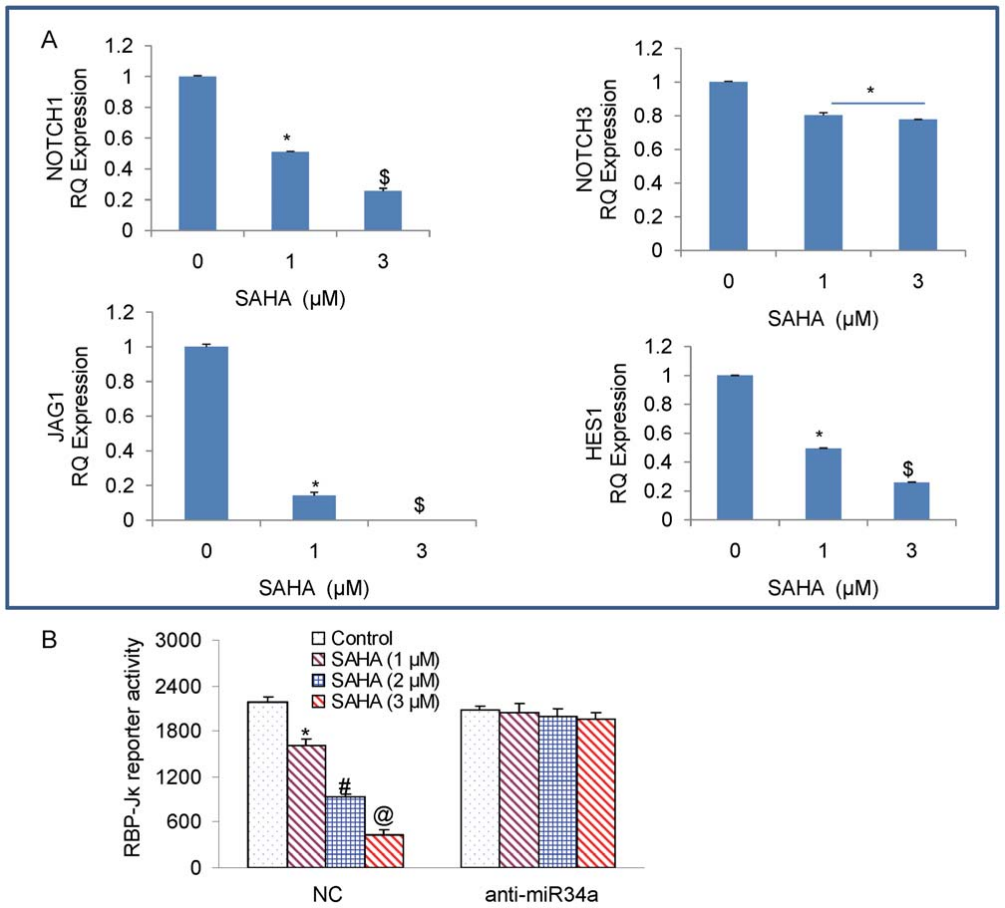
Inhibtion of self-renewal genes that are targets of miR-34a in pancreatic CSCs. (A), Pancreatic CSCs were treated with SAHA (1 and 3 µM) for 24 h, and the expression of Notch1, JAGGED1, NOTCH3 and HES1 was measured by qRT-PCR. Data represent mean ± SD. * or $  =  significantly different from control, P<0.05. (B), RBP-Jκ reporter activity. Pancreatic CSCs were transfected with either negative control (scrambled) or anti-miR34a oligonucleotides along with RBP-Jκ-LUC construct, and treated with SAHA (0–3 µM) for 24 h. Luciferase activity was measured as per manufacturer's instructions (Promega). Data represent mean ± SD. *, # or @  =  significantly different from control, P<0.05.

Notch signaling has been shown to play a role in the stem cell renewal and cell fate determination in neural, hematopoietic and embryonic stem cells [38]. Jagged 1 expression on progenitor cells induces self renewal of stem cells due to Notch signaling activation [39]. Notch receptors, ligands as well as downstream targets have been identified to be upregulated in preneoplastic lesions to invasive pancreatic cancers in human and mice, suggesting that Notch signaling may be an early event leading to accumulation of undifferentiated precursor cells in pancreatic cancer. We demonstrate that re-enforced expression of miR-34a in pancreatic CSCs upon treatment with SAHA, causes an inhibition in the mRNA expression of all the components of Notch pathway, Notch receptor Notch1, Notch 3 and its ligand Jagged1 and downstream Notch target gene Hes1 expression (Fig. 5A). Down regulation of Notch may thus contribute to the inhibition of stem cell renewal and the invasive capacity of pancreatic CSCs.

Since SAHA inhibited the expression of several components of the Notch pathway, we next measured the RBP-Jκ reporter activity by luciferase assay (Fig. 5B). SAHA inhibited the RBP-Jκ-Luciferase activity in a dose-dependent manner. By comparison, anti-miR34a blocked the inhibitory effects of SAHA on RBP-Jκ-luciferase activity. These data suggest that up-regulation of miR-34a by SAHA may have functional significance on the regulation of VEGF.

### Effect of miR-34a restoration on epithelial-mesenchymal transition of pancreatic CSCs

**Figure 6:**
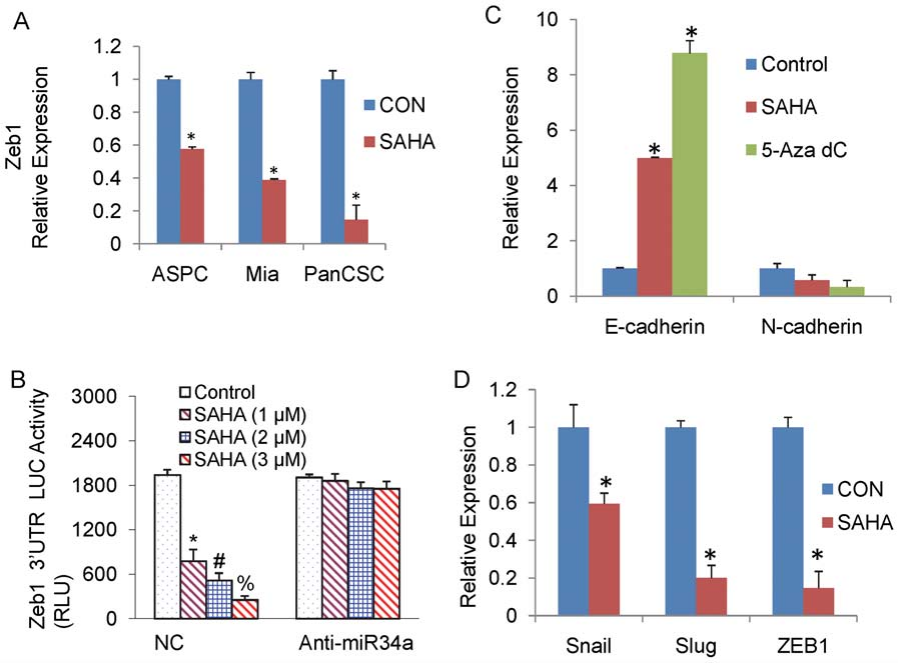
Role of miR-34a restoration on the EMT regulation in pancreatic cancer cells and CSCs. (A), Pancreatic cancer cell lines [ASPC-1(p53wt) and MiaPACA-2 (p53mutant)] and pancreatic CSCs were treated with SAHA (1 µM) for 24 h, and the expression of Zeb1 was measured by qRT-PCR. Data represent mean ± SD. *  =  significantly different from control, P<0.05. (B), Pancreatic CSCs were treated with SAHA (1 µM) for 24 h and the expression of Snail, Slug and Zeb1 was measured by qRT-PCR. HK-GAPD was used as the endogenous normalization control. Data represent mean ± SD. *  =  significantly different from control, P<0.05. (C), Regulation of Zeb-1 3′UTR reporter activity. Pancreatic CSCs were transfected with either negative control (scrambled) or anti-miR34a oligonucleotides along with Zeb1 3′UTR-Luciferase construct, and treated with SAHA (0–3 µM) for 24 h. Luciferase activity was measured as per manufacturer's instructions (Promega). Data represent mean ± SD. *, #, %  =  significantly different from control, P<0.05. (D), Pancreatic CSCs were treated with SAHA (1 µM) or 5-Aza-dC (2 µM) for 24 h and the expression of E-cadherin and N-cadherin was measured by qRT-PCR. Data represent mean ± SD. *  =  significantly different from control, P<0.05.

EMT induction in cancer cells results in the acquisition of invasive and metastatic properties [40]. We therefore investigated the role of miR-34a restoration in the EMT regulation in pancreatic CSCs. Zeb-1 is a crucial EMT activator and suppresses the expression of basement membrane components and cell polarity factors thereby promoting metastasis of tumor cells. Zeb-1 transcription was significantly inhibited in pancreatic CSCs and cancer cell lines upon treatment with SAHA (Fig. 6A and B). We next investigated the effect of SAHA on the expression of other EMT transcription factors Snail and Slug in pancreatic CSCs. Taken together, SAHA resulted in inhibition of Snail, Slug and Zeb1 transcription (Fig. 6B), thereby suggesting a role of miR-34a in the reversal of the EMT transition in pancreatic CSCs. Interestingly, 5-Aza-dC also inhibited the transcription of Slug (data not shown), which may possibly play a role in inhibition of invasion.

Since SAHA inhibited the expression of Zeb-1 through up-regulation of miR34a, we also examined the effects of SAHA on Zeb1 3′UTR-luciferase reporter activity. As demonstrated in Fig. 6C, SAHA inhibited Zeb-1 3′UTR-Luciferase activity in a dose-dependent manner. By comparison, anti-miR34a blocked the inhibitory effects of SAHA on Zeb-1 3′UTR-Luciferase activity. These data suggest that up-regulation of miR-34a by SAHA may have functional significance on EMT regulation by inhibiting Zeb-1.

Cadherin switch has been shown to occur during EMT regulation. Treatment of pancreatic CSCs with SAHA and 5-Aza-dC alone induced the expression of E-Cadherin and inhibited the expression of N-Cadherin, thereby showing that miR-34a is a strong inducer of an epithelial phenotype (Fig. 6D).

### 5-Aza-dC and SAHA inhibit migration, colony formation, invasion and spheroid formation

**Figure 7:**
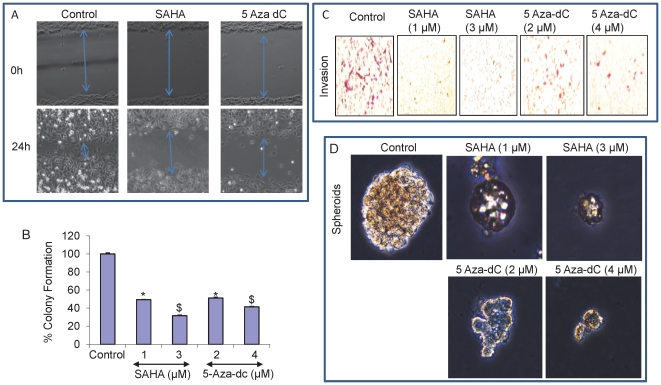
SAHA and 5-Aza-dC inhibit migration, colony formation, invasion and spheroid formation. (A), Photomicrographs demonstrating the results of the in vitro migration of pancreatic CSCs using the simple scratch technique. Pancreatic CSCs were grown in monolayer, scratched and treated with SAHA and 5-Aza-dC for 24 h. (B), SAHA and 5-Aza-dC inhibit colony formation by CSCs. Pancreatic CSCs were seeded in soft agar and treated with SAHA (1 and 3 µM) or 5-Aza-dC (2 and 4 µM) for 21 days. At the end of incubation period, colonies were counted. Data represent mean ± SD. * and $  =  significantly different from control, P<0.05. (C), CSCs were plated onto the Matrigel-coated membrane in the top chamber of the transwell and treated with SAHA (1 and 3 µM) or 5-Aza-dC (2 and 4 µM) for 48 hrs. Cells invaded to the lower chambered were fixed with methanol, stained and photographed. (D), Pancreatic CSCs were seeded in suspension and treated with SAHA (1 and 3 µM) or Aza-5dC (2 and 4 µM) for 7 days. Pictures of spheroids formed in suspension were taken by a microscope.

To further understand the effect of up-regulation of miR-34a expression on the invasive potential of pancreatic CSCs, we next investigated the effect of 5-Aza-dC and SAHA on the pancreatic CSCs using scratch migration, soft agar colony formation, and transwell boyden chamber invasion assays. Representative microphotographs of cells invading through scratch generated for the control and 5-Aza-dC and SAHA treated pancreatic CSCs showed significantly less migration in the treated group panel as compared to control group (Fig. 7A).

>5-Aza-dC and SAHA reduced the ability of pancreatic CSCs to form soft agar colonies as compared to control (Fig. 7B) and transfection with anti-miR-34a nullified the effects of 5-Aza-dC and SAHA on colony formation (data not shown). Invasion of pancreatic CSCs through the matrigel coated membrane showed that the number of invading cells was significantly inhibited in 5-Aza-dC and SAHA treated cells and was approximately 75% less than the number of cells invading in the control group (Fig. 7C). Further, 5-Aza-dC and SAHA inhibited the spheroid formation in pancreatic CSCs as seen in Fig. 7D. These results demonstrate that miR-34a might play an important role in the regulation of pancreatic tumorigenesis by inhibiting the self-renewal capacity of pancreatic CSCs, metastasis and invasion.

## Discussion

MicroRNAs (miRNAs) are small non-coding RNAs that regulate the expression of other genes by transcriptional inhibition or translational repression. Accumulating evidence suggests a role for microRNAs in human carcinogenesis as novel types of tumor suppressors or oncogenes [41], [42]. Recently, miR-34a family members were found to be directly regulated by TP53, and the functional activity of miR-34a indicated a potential role as a tumor suppressor. We demonstrate in this study that miR-34a expression is down regulated in human pancreatic CSCs and pancreatic tumor derived cell lines, irrespective of their p53 mutational status. 5Aza-dC and SAHA can epigenetically restore the expression of miR-34a independent of the p53 mutational status in pancreatic cancer. Furthermore, miR-34a inhibits SIRT1 expression through a miR-34a-binding site within the 3′ UTR of SIRT1. This finding substantiates our hypothesis that miR-34a may be thus epigenetically regulated in pancreatic cancer.

DNA methylation is involved in regulating normal biologic processes as well as carcinogenesis. DNA methylation is a heritable modification of the DNA structure, which do not alter the specific sequence of base pairs responsible for encoding the genome, but which can directly inhibit gene expression. Hypomethylating agents (azacitidine and decitabine) and Histone deactylase (HDAC) inhibitors suberoylanilide hydroxamic acid (SAHA) have been approved by FDA for the treatment of myelodydplastic syndrome and cutaneous T cell lymphoma, respectively. Preclinically and clinically these agents have also shown to sensitize cancer cells to chemotherapeutic agents and biologically targeted drugs [43], [44]. Novel therapeutic regimens can be developed by modulating histone acetylation along with other therapeutic strategies such as inhibition of DNA methylation, intracellular signal transduction pathway and transcription factors as well as induction of apoptosis.

Our data reveal that the chromatin modulators, 5Aza-dC and SAHA can epigenetically restore the expression of miR-34a independent of the p53 mutational status in pancreatic CSCs and pancreatic cancer tumor derived cell lines. These chromatin modifying agents can also regulate the downstream targets of miR-34a, thus suggesting the role of miR-34a restoration in regulating the pancreatic tumorigenesis and providing a functional basis for the epigenetic inactivation of this miRNA in pancreatic cancer. Another plausible explanation of this could be that in addition to the epigenetic silencing of miR-34a, SIRT1 a downstream target of miR-34a may be a key player through a SIRT1-p53 pathway.

SIRT1 is up regulated in pancreatic cancer, which is a NAD dependent deactylase and has been shown to inhibit several pro-apoptotic genes. SIRT1 inactivates p53 by deacetylation of p53 [45]. miR-34a is itself a transcriptional target of p53 suggesting a positive feedback between p53 and miR-34a. SAHA down regulates the expression of SIRT1 through upregulation of miR-34a, which can now participate in a positive feedback loop leading to further activation of p53. The microRNA mediated feed forward loop stabilizes p53 through acetylation, leading to increased transcriptional activity of p53, thus promoting apoptosis. This is represented from our data, which shows induction of acetylated p53 and its transcriptional targets p21^WAF1^ and PUMA that regulate the cell cycle and apoptosis, upon treatment with SAHA. Thus it explains how epigenetic silencing of miR-34a would interrupt this feedback, resulting in lower p53 activity, thereby providing a selective advantage to the pancreatic cancer cells. These observations are in concert with published studies of Yamakuchi et al [46], where they have shown that p53 acetylation on lysine 382 is increased after ectopic miR-34a expression and also by Fujita et al who have reported the down-regulation of SIRT1 by miR-34a [47].

Deregulated Notch signaling is associated with pancreatic tumorigenesis [48], [49] and cell invasion [50]. Up regulation of Zeb-1 and Jagged 1 has been associated with the aggressiveness of pancreatic adenocarcinoma resulting in increased Notch signaling [39]. We have demonstrated here that re-expression of miR-34a in pancreatic cancer stem cells on treatment with SAHA, causes an inhibition in the mRNA expression of all the components of Notch pathway. Down regulation of Notch contributes to the inhibition and apoptosis of pancreatic CSCs. Interestingly, SAHA also inhibited the expression of VEGF thus suggesting that by inhibiting the Notch pathway, miR-34a may play an anti-angiogenic and anti-invasive role as well. Restoration of miR-34a expression in pancreatic CSCs by the chromatin modifiers significantly reduced *in vitro* migration, invasion and anchorage-independent growth.

In conclusion, we demonstrate that miR-34a is a tumor suppressor gene which targets multiple critical oncogenic pathways. The restoration of miR-34a by pharmacological intervention thus provide novel prospects for clinical innovation laying the ground work for *in vivo* experiments in the future. The effect of these chromatin modulators on xeno-transplants of pancreatic CSCs in mice will provide us with an insight of the future of tumor suppressor miRNAs restorations and their physiological significance.

## Methods

### Reagents

Antibodies against p21^/WAF1/CIP1^, p27^/KIP1^, cyclin D1, CDK2, CDK4 and CDK6 were purchased from Cell Signaling Technology, Inc. (Danvers, MA). Antibodies against Bcl-2, SIRT1, VEGF, acetylated and total p53 and PUMA and β-actin were purchased from Santa Cruz Biotechnology Inc. (Santa Cruz, CA). 5-Aza-dC was purchased from Calbiochem. SAHA was obtained from the National Cancer Institute. Penicillin, streptomycin, RPMI-1640 medium, Keratinocyte and fetal bovine serum (FBS) were obtained from Invitrogen Corporation (Carlsbad, CA). Tris-HCl, glycine, sodium chloride, sodium dodecyl sulfate (SDS) and bovine serum albumin (BSA) were obtained from Sigma-Aldrich (St. Louis, MO). Annexin-V-FITC/propidium iodide (PI) kit was purchased from BD Biosciences (San Jose, CA). Enhanced chemiluminescence (ECL) Western blot detection reagents were from Amersham Life Sciences Inc. (Arlington Heights, IL).

### Cell culture

MIA PaCa-2 and AsPC-1 cells were obtained from the American Type Culture Collection (Manassas, VA). Human pancreatic CSCs (CD44+/CD24+/ESA+) have been characterized and described previously [17]. CSCs were cultured in DMEM supplemented with 1% N2 Supplement (Invitrogen), 2% B27 Supplement (Invitrogen), 20 ng/ml human platelet growth factor (Sigma-Aldrich), 100 ng/ml epidermal growth factor (Invitrogen) and 1% antibiotic-antimycotic (Invitrogen) at 37°C in a humidified atmosphere of 95% air and 5% CO2.

### Isolation of RNA

The total RNA was isolated from the pancreatic cancer stem cells and pancreatic cell lines using TRIzol (Life Technologies) according to the manufacturer's instructions. The RNA pellets were then frozen and stored at −80°C until use.

### Evaluation of miRNAs and mRNA expression levels by quantitative real time-PCR

qRT-PCR of microRNAs was performed using TaqMan microRNA assays (Applied Biosystems) in an Applied Biosystems 7300 Sequence Detection System (Applied Biosystems, Foster City, CA). Ten nanograms of total RNA were reverse transcribed using a TaqMan® MicroRNA Reverse Transcription (RT) kit from Applied Biosystems. Each RT reaction contained 1x stem-loop RT specific primer, 1x reaction buffer, 0.25 mM each of dNTPs, 3.33 U/µl Multiscribe RT enzyme and 0.25 U/µl RNase inhibitor. The 15-µl reactions were incubated for 30 min at 16°C, 30 min at 42°C, and 5 min at 85°C and then held at 4°C. The PCR reaction was performed using a standard TaqMan® PCR kit protocol (Applied Biosystems). Briefly, following the RT step, 1.33 µl of the RT reaction were combined with 1 µl of a TaqMAn MicroRNA Assay (20x; forward primer, reverse primer and probe) and 17.67 µl of TaqMan® Universal PCR Master Mix, No AmpErase® UNG in a 20 µl final volume. The reactions were incubated at 95°C for 10 min, followed by 40 cycles of 95°C for 15 s and 60°C for 1 min. The expression of miR-34a was normalized against the expression of another small RNA, RNU48 as endogenous normalization control. All assays were performed in triplicate and were calculated on the basis of ΔΔ*C*t method. The n-fold change in miRNAs expression was determined according to the method of 2^−ΔΔCT^.

For the quantification of gene amplification, Real-time PCR was performed using an ABI 7300 Sequence Detection System in the presence of SYBR- Green. Briefly, RNA isolated with or without treatment with the chromatin modifiers using TRIzol (Life Technologies) were reverse transcribed. cDNA reactions were amplified with QPCR SYBR Green Mix (Applied Biosystems) and the following gene-specific primers:

NOTCH 1 (5′- gga cct cat caa ctc aca cg -3′, 5′- ggt gtc tcc tcc ctg ttg tt -3′)

NOTCH 3 (5′- gga cat gtt cca tag cct tg -3′, 5′- tcc cac att tac agg gac ac -3′)

JAG (5′- ggc ctc tga aga aca gaa ca -3′, 5′- ttt ctc aat ggg gtt ttt ga -3′)

HES1 (5′- acc aaa gac agc atc tga gc -3′, 5′- ggt gct tca ctg tca ttt cc -3′)

SNAIL (5′- acc cca cat cct tct cac tg -3′, 5′- tac aaa aac cca cgc aga ca -3′)

SLUG (5′- aca cac aca cac cca cag ag -3′, 5′- aaa tga ttt ggc agc aat gt -3′)

ZEB1 (5′- gca caa cca agt gca gaa ga -3′, 5′- cat ttg cag att gag gct ga -3′)

E- CADHERIN (5′- tgc tct tgc tgt ttc ttc gg-3′, 5′- tgc ccc att cgt tca agt ag-3′)

N- CADHERIN (5′- tgg atg gac ctt atg ttg ct -3′, 5′- aac acc tgt ctt ggg atc aa -3′)

GAPDH (5′- gag tca acg gat ttg gtc gt -3′, 5′- ttg att ttg gag gga tct cg -3′)

Target sequences were amplified using incubated at 95°C for 10 min, followed by 40 cycles of 95°C for 15 s and 60°C for 1 min. HK-GAPD is used as endogenous normalization control. All assays were performed in triplicate and were calculated on the basis of ΔΔ*C*t method. The n-fold change in miRNAs expression was determined according to the method of 2^−ΔΔCT^.

### Transfecting cells with pre-miRs™ and anti-miRs

Enhanced or knockdown expressions of miR-34a were performed by transfection with miR-34a precursor (Ambion) or anti-miR-34a (Ambion), respectively. All transfections were carried out in triplicate. 50 nmol of pre-miR™, anti-miR and Negative-control pre and anti-miR 34a (Ambion), respectively, were transfected using lipofectamine 2000 (Invitrogen) for 24 h. Twenty-four hours following transfection the cells were treated in presence or absence of 5-Aza-dC or SAHA and the Total-RNA and Protein were isolated 48 hours post treatment.

### Lentiviral reporter assay

The cop-GFP and luciferase genes were cloned downstream of CSL/RBP-Jκ-response element, containing four CSL/RBP-Jκ binding motifs (pGreen Fire1-4xNotch-mCMV-EF1-Neo; System Biosciences, Mountain View, CA). For *in vitro* assays, stably transduced pancreatic CSCs were plated at 5–10,000 cells per well in 24-well plates and treated with various doses of drugs. After incubation, CSCs were analyzed for either GFP expression by fluorometer or luciferase activity by luminometer. Other reporter assays were performed in a similar manner.

### Viral production and infection

For lentiviral particle production, HEK 293T cells were transduced with plasmids of interest in the presence of lipofectamine. Viral supernatants were collected, mixed with PEG and concentrated by ultracentrifugation to produce virus stocks with titers of 1×10^8^ to 1×10^9^ infectious units per milliliter. Viral supernatant was collected for three days by ultracentrifugation and concentrated 100-fold. Titers were determined on HEK293T cells. Human pancreatic CSCs were transduced with a mixture of viral particles and polybrene with two rounds of infections.

### Measurement of apoptosis

Early apoptotic process is characterized by changes in the phospholipid bilayers of cell membranes. The phosphotidylserine (PS) component of the phospholipid bilayers are externalized and can be detected by fluorescence labeling. Annexin V is a member of the annexin family of calcium-dependent phospholipid-binding proteins which has a high affinity for PS-containing phospholipid bilayers. Staining with FITC-conjugated annexin V and propidium iodide (PI) can help quantitate subpopulations of cells with compromised membrane integrity. Untreated control and treated cells were washed twice with cold PBS and resuspended in buffer at a concentration of 10^6^ per ml. Cells were mixed with 10 µl of fluoresceine isothiocyanate (FITC)-conjugated annexin V reagent and 10 µl of 3 mM propidium iodide (PI). After a 15 min incubation at room temperature in the dark and further washings, samples were analysed by flow cytometry. Flow cytometry was performed with a FACScan analyzer (Becton Dickinson) with 15 mW argon ion laser (488 nm) and Cell Quest software. Annexin V staining was detected in the FL1 (green) channel, whereas PI staining was monitored in the FL2 (red) channel: appropriate quadrants were set and the percentage of cells negative for both stains (viable cells), positive for annexin V (apoptotic cells) and positive for PI (dead cells) were acquired.

### Cell cycle distribution analysis

Cell cycle distribution and ploidy status of pancreatic cancer cells after treatment with Aza-5-dC and SAHA and untreated controls were determined by flow cytometry DNA analysis as described previously [51], [52] . Briefly at the end of treatments, cells were detached from the plates by the addition of 0.25% trypsin, washed in PBS, fixed in 70% ethanol at 4°C and treated with 10 mg/ml RNAse for 30 min at 37°C. The DNA content was evaluated in a FACScan flow cytometer (Becton-Dickson, NJ, USA) after staining cells with propidium iodide buffer (0.1 mM EDTA, 0.1% Triton X-100, 50 µg/ml propidium iodide, PBS pH 7.4) for 15 min in the dark at room temperature. For cell cycle analysis, only single cells were considered. A pass filter of 585 nm was used to collect PI fluorescence, acquiring 10,000 events for each sample.

### Caspase-3/7 assay

Pancreatic cancer stem cells (3×104 per well) were seeded in a 96-well plate with 200 µl culture medium. Approximately 16 h later, cells were treated with and without various doses of 5Aza-dC and SAHA. Casapse-3/7 activity was measured by a fluorometer as per manufacturer's instructions (Invitrogen).

### Western blot analysis

At the end of treatments with Aza-5-dC and SAHA, as well as untreated control were analyzed using western blot analysis as described before [17]. Breifly, cells were washed with cold PBS and lysed in ice-cold lysis buffer (50 mM Tris-HCl pH 7.5, 2 mM EDTA, 2 mM EGTA, 10 mM β-glycerophosphate, 150 mM NaCl, 0.5% NP-40, 1 mM phenyl-methyl sulfonyl fluoride, 1 mM NaF, 1 mM DTT, 1% β-mercaptoethanol and 4 µg/µl complete protease inhibitor cocktail (EMD Biosciences). Cell lysates were centrifuged at 15,000 g for 15 min at 4°C and protein concentration was determined in the supernatants using the Coomassie Plus protein assay reagent (Pierce, Rockford, IL) using bovine serum albumin as standard. About 40 µg of crude proteins were mixed with SDS sample buffer, denatured and electrophoresed in 12% SDS-PAGE gels. After electrophoresis, gels were transferred to nitrocellulose membranes by electroblotting and blocked for two h at room temperature in TBST (50 mM Tris-HCl pH 7.5, 150 mM NaCl, 0.2% Tween-20) containing 5% nonfat milk. Blots were sequentially incubated with the primary and secondary antibodies, washed in TBS-T. Membranes were developed by enhanced chemiluminescence (ECL-Plus, Amersham Pharmacia Biotech, Piscataway, NJ) and exposed to Kodak Biomax Light films for 1–10 min. In order to detect a second protein, some blots were stripped by incubation with 100 mM Tris-HCl, pH 7.4, 100 mM β-mercaptoethanol, and 2% SDS at 60°C for 30 min.

### Motility assay

Scratch migration assay was used to study the horizontal movement of cells. A confluent monolayer of cells was established and then a scratch is made through the monolayer, using a standard 1–200 µl plastic pipet tip, which gives rise to an in vitro wound, washed twice with PBS, and replaced in media with or without chromatin modifying agents. Cancer stem cells migrate into the scratch area as single cells from the confluent sides. The width of the scratch gap is viewed under the microscope in four separate areas each day until the gap is completely filled in the untreated control wells. Three replicate wells from a 6-well plate were used for each experimental condition.

To quantitate vertical motility, an in vitro transwell invasion assay was used. In this assay, 1×10^5^ cells were plated in the top chamber onto the Matrigel coated Membrane (24-well insert; pore size, 8 µm; Corning Costar). Each well was coated freshly with Matrigel (60 µg; BD Bioscience) before the invasion assay. Cells were plated in complete medium and medium supplemented with chromatin modifying agents was used in the lower chamber. The cells were incubated for 48 hours and cells that did not invade through the pores were removed by a cotton swab. Cells on the lower surface of the membrane were fixed with methanol and stained with crystal violet. The number of cells invading through the membrane was counted under a light microscope (40X, three random fields per well).

### Soft agar colony assay for assessment of tumorigenic potential *in vitro*

To examine the anchorage independent growth, the pancreatic cancer stem cells derived from primary human tumors were suspended (10^3^ cells/ml) in 2 ml of 0.3% agar with 1% N2 Supplement (Invitrogen), 2% B27 Supplement (Invitrogen), 20 ng/ml human platelet growth factor (Sigma-Aldrich), 100 ng/ml epidermal growth factor (Invitrogen) and 1% antibiotic-antimycotic (Invitrogen) overlaid into six-well plates containing a 0.5% agar base. All samples were plated in triplicate. Colonies with >0.2 mm in diameter were counted on day 21. Colonies were stained with 0.001% crystal violet blue and counted.

### Statistical analysis

The mean and standard deviation (SD) were calculated for each experimental group. Differences between groups were analyzed by one or two way ANOVA using PRISM statistical analysis software (GrafPad Software, Inc., San Diego, CA). Significant differences among groups were calculated at P<0.05.
